# Raising awareness of bronchiectasis in primary care: overview of diagnosis and management strategies in adults

**DOI:** 10.1038/s41533-017-0019-9

**Published:** 2017-03-13

**Authors:** James D. Chalmers, Sanjay Sethi

**Affiliations:** 1 Scottish Centre for Respiratory Research, University of Dundee, Dundee, UK; 2 University at Buffalo, State University of New York, Buffalo, NY USA

## Abstract

Bronchiectasis is a chronic lung disease characterised by recurrent infection, inflammation, persistent cough and sputum production. The disease is increasing in prevalence, requiring a greater awareness of the disease across primary and secondary care. Mild and moderate cases of bronchiectasis in adults can often be managed by primary care clinicians. Initial assessments and long-term treatment plans that include both pharmacological and non-pharmacological treatments, however, should be undertaken in collaboration with a secondary care team that includes physiotherapists and specialists in respiratory medicine. Bronchiectasis is often identified in patients with other lung diseases, such as chronic obstructive pulmonary disease, asthma, and in a lesser but not insignificant number of patients with other inflammatory diseases, such as rheumatoid arthritis and inflammatory bowel disease. Overall goals of therapy are to prevent exacerbations, improve symptoms, improve quality of life and preserve lung function. Prompt treatment of exacerbations with antibiotic therapy is important to limit the impact of exacerbations on quality of life and lung function decline. Patient education and cooperation with health-care providers to implement treatment plans are key to successful disease management. It is important for the primary care provider to work with secondary care providers to develop an individualised treatment plan to optimise care with the goal to delay disease progression. Here, we review the diagnosis and treatment of bronchiectasis with a focus on practical considerations that will be useful to primary care.

## Introduction

**Fig. 1:**
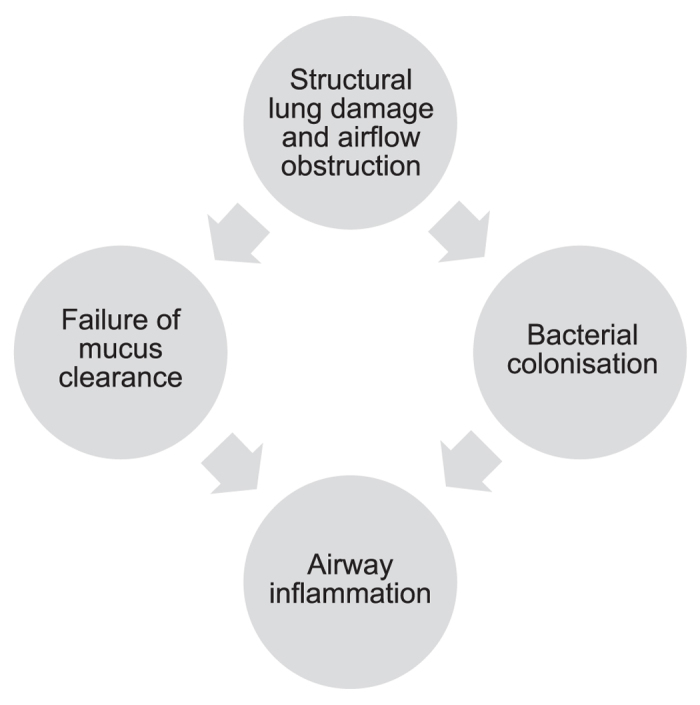
Vicious cycle hypothesis of bronchiectasis^4, 5^

Non-cystic fibrosis bronchiectasis (referred to as bronchiectasis throughout) is a chronic lung disease characterised by recurrent infection, inflammation, persistent cough and production of sputum.^1, 2^ Bronchiectasis results from permanent dilation of the airways.^3^ The primary insult is often unknown, but pathological changes in response to Cole’s vicious cycle hypothesis^4, 5^ are thought to be responsible for disease progression (Fig. 1).

Causes may include post-infective injury (previous bacterial or viral infections), congenital defects of the mucociliary clearance such as primary ciliary dyskinesia (PCD) or immune deficiency.^1, 2, 5, 6^ A listing of possible causes is shown in Table 1, although often the underlying cause is undetermined.^1, 2, 7–14^ Where the cause is not identified, patients are classified as having ‘idiopathic’ bronchiectasis. Because prior infections are common causes of bronchiectasis,^2, 15^ recent guidelines suggest asking patients about previous respiratory infections, including previous tuberculosis, to establish possible linkage with the onset of bronchiectasis symptoms.^2^ In Lady Windermere syndrome,^16, 17^ a syndrome named for a character with a chronic voluntarily suppressed cough in the Oscar Wilde play *Lady Windermere’s Fan*,^18^ bronchiectasis is thought to be caused by chronic *Mycobacterium avium* complex or other pulmonary non-tuberculous mycobacteria (NTM) infection.^19^ It is more prevalent in tall, lean, middle-aged women than it is in the general population.^19^

**Table 1:** Possible causes and associated comorbid conditions of non-cystic fibrosis bronchiectasis

Causes	Frequency (%)
Primary cause	
Undetermined (idiopathic bronchiectasis)	30–53
Previous infection–bacterial or viral	33–42
Aspiration/inhalation injury	2–4
Congenital defect of large airway (e.g., Mounier-Kuhn syndrome)	<1
Immune deficiency (hypogammaglobulinaemia)	1–8
Primary ciliary dyskinesia	1–17
Connective tissue disease/rheumatoid arthritis/Sjögren’s syndrome/systemic sclerosis	3–6
Cause or comorbid condition	
COPD^a^	4–69
Asthma^a^	17.5–43.0
Allergic bronchopulmonary aspergillosis (associated with asthma)	1–7
Inflammatory bowel disease	1–2
Non-tuberculosis mycobacterial infection	0.7–34.0

Data from Aliberti *et al*.,^8^Agusti *et al*.,^7^Chalmers and Hill,^1^ Fowler *et al*.,^9^ Gupta *et al*.,^10^ Ni *et al*.,^11^ Park and Olivier,^12^ Pasteur *et al*.,^2^ Quint *et al*.^13^

*COPD* chronic obstructive pulmonary disease

^a^Whether COPD and asthma are the underlying cause of bronchiectasis, or are associated conditions, is often not clear. Non-tuberculous Mycobacteria and allergic bronchopulmonary aspergillosis are thought to be both causes and consequences of bronchiectasis

Bronchiectasis can also result from rare congenital defects such as PCD, in which the epithelial cell motor cilia are dysfunctional, resulting in mucus accumulation.^20, 21^ Airway obstruction from the mucus and subsequent inflammation and bacterial infection contribute to development of bronchiectasis.^20, 21^ Other rare genetic causes associated with bronchiectasis have recently been reviewed in detail,^22^ and include Williams-Campbell syndrome (a cartilage deficiency), Mounier-Kuhn syndrome (tracheobronchomegaly), common variable immune deficiency (hypogammaglobulinaemia), inherited connective tissue disorders, α1-antitrypsin deficiency and yellow nail syndrome.

Bronchiectasis prevalence estimates vary by region and increase with age but suggest that bronchiectasis is a relatively common disease. Prevalence estimates in the United States, United Kingdom and New Zealand are variable owing to differences in diagnostic techniques and methods^2, 13, 23–26^; recent estimates range from 370 per 100,000 persons to 566 per 100,000 persons.^13, 24^ These estimates also may appear to be increasing owing to improved diagnosis and recognition, including wider use of high-resolution chest computed tomography (HRCT) scans.^13, 27^ To put the prevalence in context, these estimates suggest there may be 1 patient with bronchiectasis for every 20 patients with chronic obstructive pulmonary disease (COPD) in Western countries.

Bronchiectasis is commonly found in patients with a diagnosis of COPD^11^ and asthma.^28, 29^ Coexistence of bronchiectasis with HIV, rheumatoid arthritis, inflammatory bowel disease and pulmonary fibrosis also has been shown.^13, 28^ A high awareness of this overlap is needed for primary care physicians to identify patients with bronchiectasis, as symptoms can be easily dismissed as part of the underlying disorder.

The aim of this manuscript is to provide a focused review of bronchiectasis and its management in adult patients for the general practitioner or primary care physician. Guidelines for treatment of bronchiectasis are available from the British Thoracic Society,^2, 30^ the Thoracic Society of Australia and New Zealand^25, 31^ and SEPAR (Spain),^32^ but not in the United States. This review considers these guidelines, along with newly published information.

## Clinical presentation—when to suspect bronchiectasis

*A 66-year-old lady presents with a chest infection associated with cough productive of green sputum and increasing shortness of breath. She had never smoked and has no relevant past medical history. Chest x-ray shows no abnormality. She is treated with antibiotic therapy by her primary care physician and improves. She attends again a few months later with worsening productive cough. Her primary care physician notes that she has had several courses of antibiotics for chest infections over the previous 3 winters and has reported a chronic productive cough on a daily basis for the past 3 years.*

In new patients or those that do not have an established diagnosis, one of the most common core symptoms is a persistent cough (>90% of patients), often producing large quantities of mucoid (white or clear) or purulent (dark yellow, green or brown) sputum^2^ (Table 2). Adults may have a history of symptoms over many years. Recurrent respiratory tract infections also raise the possibility of bronchiectasis, and patients may take a long time to recover from chest infections or require multiple courses of antibiotics before symptoms fully resolve. Dyspnoea is present in a high proportion of cases, with frequent haemoptysis in severe disease. These symptoms can be variable across patients, with some having symptoms daily and others only having symptoms during exacerbations.^5^ The longstanding textbook teaching of bronchiectasis patients with widespread crackles, digital clubbing and cachexia is now rarely seen.

**Table 2:** Symptoms/signs of bronchiectasis

Clinical signs of bronchiectasis
Core symptoms
Persistent cough
Sputum production
Breathlessness on exertion
Recurrent pneumonia/lung infections/bronchitis
Asthma or COPD unresponsive to usual treatment
Additional signs and symptoms
Coarse crackles on auscultation (often absent)
Chronic rhinosinusitis
Chest discomfort
Fatigue and weight loss
Signs associated with underlying disorders (e.g., rheumatoid arthritis, yellow nail syndrome, connective tissue disease)

Pasteur *et al*.^2^ and Chalmers *et al*.^5^

*COPD* chronic obstructive pulmonary disease

Diagnosis may be difficult when a patient has already received a diagnosis of another chronic respiratory disease such as COPD or asthma. Furthermore, considerable diagnostic confusion exists between bronchiectasis, asthma and COPD. Patients with primary bronchiectasis are often first labelled as asthma and COPD. Further adding to this complexity is the occurrence of secondary bronchiectasis in patients with asthma and COPD, and the coexistence of these common disorders in the same patient. A thorough clinical evaluation is essential and often the best diagnostic tool to determine the primary condition/s and manage accordingly. Bronchiectasis should be suspected in patients when there is a poor response to standard therapy, when unusual pathogens are isolated from sputum or when patients do not have a typical clinical history of COPD (e.g., absence of smoking history or young age of onset). In addition, patients with asthma may develop bronchiectasis associated with an immunological reaction to *Aspergillus*, known as allergic bronchopulmonary aspergillosis (ABPA).^2^ Such patients present with a history of asthma that is poorly controlled, often with the production of large volumes of sputum or plugs. Therefore, bronchiectasis and ABPA should be considered as a potential contributor to severe asthma or poor asthma control.

## How to diagnose bronchiectasis

### Primary care

The majority of respiratory tract infections seen in the primary care setting are self-limiting and do not require further investigation. In addition, the majority of patients with a chronic cough will not have bronchiectasis. In one study of 266 patients with chronic cough lasting longer than 8 weeks referred to a secondary care cough clinic and who completed follow-up, most patients had positive outcomes and did not receive a bronchiectasis diagnosis.^33^ The largest group of patients (29%) had asthma that was demonstrated by bronchodilator reversibility. Gastro-oesophageal reflux disease (GORD) related cough was noted in 22% and most of these patients were sensitive to proton pump inhibitor treatment. Angiotensin converting enzyme inhibitor (ACEi)-induced cough was present in 14% and resolved on withdrawal of the ACEi. Only one patient had a diagnosis of bronchiectasis. Indicators of possible bronchiectasis are sputum production, which is often absent with GORD, cough variant asthma or cough hypersensitivity, and episodes of respiratory tract infections, which are also uncommon with these disorders. Fevers or night sweats with a chronic cough are unusual in bronchiectasis and suggest pulmonary tuberculosis or pulmonary non-tuberculous Mycobacterial disease in the appropriate clinical context.

Bronchiectasis should be considered as a possible diagnosis where respiratory tract infections are severe, persistent, unusual or recurrent (represented by the helpful acronym SPUR). Patients with suspected clinical signs and symptoms of bronchiectasis should be evaluated by a thorough clinical examination to rule out other possible causes and a sputum sample should be sent for microbiological analysis.^2, 25^

*Haemophilus influenzae, Pseudomonas aeruginosa, Streptococcus pneumoniae* and *Moraxella catarrhalis* are among the most common pathogens isolated from patients with bronchiectasis.^2, 34, 35^ Standard bacterial cultures will not identify some important bronchiectasis-associated pathogens such as NTM, but these can be excluded by requesting specific cultures for Mycobacteria. Negative sputum cultures do not exclude a diagnosis of bronchiectasis. Sending samples when patients are clinically ‘well’ is important, as culture positive samples with chest infections are common in many conditions, but a culture positive sputum sample when the patient is well increases the likelihood that the patient has bronchiectasis.

Chronic colonisation with *P. aeruginosa* occurs in many patients and is associated with more severe disease.^2, 36^ In a recent systematic review,^37^ which included a meta-analysis of 21 studies, mortality for bronchiectasis patients with *P. aeruginosa* colonisation was higher (pooled odds ratio of 2.95, *P* < 0.0001) than in patients without colonisation. In patients with *P. aeruginosa*, mortality was 7.7% at 1 year and 30 to 50% at 5 years.^37^ In contrast, mortality for bronchiectasis patients without *P. aeruginosa* was 0% at 1 year and 9 to 15% at 5 years. Hospital admission rates were significantly increased in those with *P. aeruginosa,* as were exacerbation rates (those with *P. aeruginosa* infection had an average of 1 additional exacerbation per patient per year than those without). *P. aeruginosa* was also associated with a worsened quality of life.^37^ Isolation of *P. aeruginosa* in a new patient or for the first time in a patient with recurrent respiratory tract infections should be promptly referred to secondary care for antibiotic treatment.^2^

Knowing which pathogen is present, if any, will help determine the most effective antibiotic treatment. However, isolation of a pathogen does not require treatment if the patient is well, as many patients are chronically infected with organisms that will not be eradicated by repeated short courses of oral antibiotics.^6, 38^

**Fig. 2:**
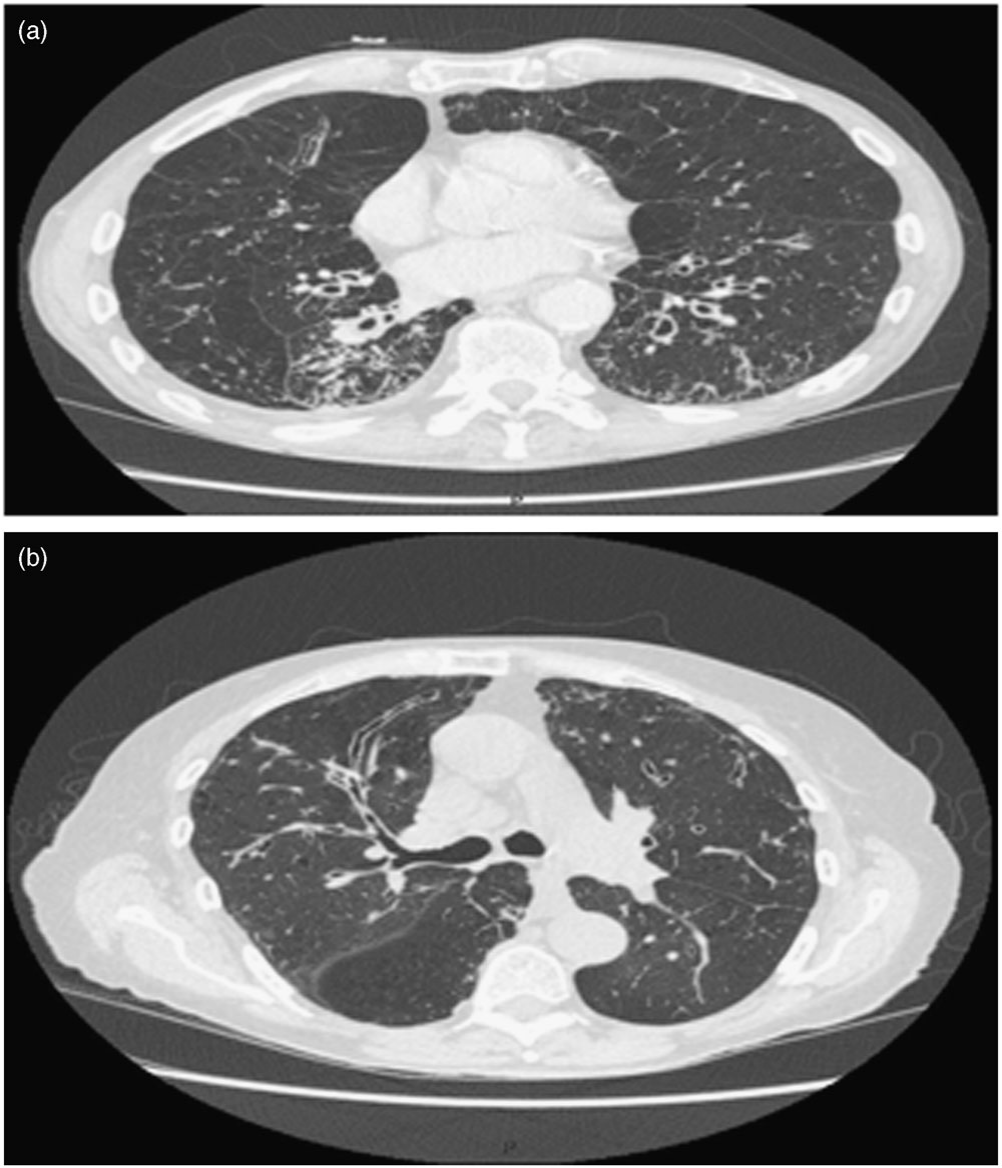
Example high-resolution chest computed tomography images of bronchiectasis. **a** Cylindrical bronchiectasis; **b** longitudinal or varicose bronchiectasis

A regular chest x-ray may be insensitive to the changes caused by bronchiectasis, as in the clinical example above.^39^ Although an HRCT scan of the chest is the radiological investigation of choice in the diagnosis of bronchiectasis,^2, 30^ it may also be identified using a standard CT scan. Bronchial dilation (luminal diameter greater than accompanying pulmonary artery/lack of tapering) is the defining feature (Fig. 2).^2, 25, 30^ Bronchial wall thickening also may be present.^2^ If HRCT is not indicative of bronchiectasis, then the diagnosis can be excluded.

In some cases, radiological evidence for bronchiectasis will be found in patients without overt symptoms. Radiological evidence of asymptomatic bronchiectasis should be investigated to determine if a causal event might explain the finding. This has been reported to occur in patients with underlying rheumatoid arthritis or humoral immune deficiency.^2^ However, it should be emphasised that bronchiectasis is a clinical diagnosis, supported by imaging, rather than a condition identified by imaging alone.

Lung function should be evaluated using spirometry,^2, 25, 30^ assessing forced expiratory volume in the first second (FEV_1_), forced vital capacity and peak expiratory flow.^2^ Although up to 80% of adult patients with bronchiectasis in secondary care will exhibit airflow obstruction, some show no reduction in airflow and spirometry may be completely normal.^2^ Presence of reduced FEV_1_ is highly predictive of mortality, hospital admission, exacerbation frequency and worse quality of life;^40^ however, it is important to emphasise that airflow reduction is a marker of severity, but it is not useful for diagnosis. For example, in one study of 608 patients with confirmed bronchiectasis, 49.5% had airflow obstruction, 18.8% had restrictive spirometry, yet 31.7% had normal spirometry.^40^

*A 71-year-old lady presents with 2 years of worsening cough, sputum production and 5 chest infections in the past year requiring antibiotics. She is an ex-smoker, has a previous history of asthma and is treated with an inhaled corticosteroid. She also has a history of rheumatoid arthritis, but has had well controlled joint disease for several years. Sputum culture is positive for H. influenzae. The primary care physician suspects bronchiectasis and refers the patients for a high resolution CT scan. This shows bilateral lower lobe bronchiectasis.*

The above example illustrates the difficulties of identifying and managing bronchiectasis in the context of multiple comorbidities. The presentation could relate to poorly controlled asthma, undiagnosed COPD, immunosuppression resulting from tumour necrosis factor antagonist therapy, Mycobacterial infection or bronchiectasis. The diagnosis of bronchiectasis is easily missed in such patients*.*

Consequently, recognition of comorbid/associated conditions associated with bronchiectasis is very important (Table 1).^2, 13, 41, 42^ Prevalence of bronchiectasis in patients with COPD has been found to range from a low of 4% (ref. 7) to as high as 69%, with mean prevalence of 54% in a recent systematic literature review.^11^ In many studies in patients with COPD, the presence of bronchiectasis was associated with increased presence of *P. aeruginosa* and other pathogenic microorganisms in sputum, reduced lung function, greater sputum production, more frequent exacerbations and increased mortality versus those with COPD alone.^11, 43^ Patients with some non-pulmonary diseases (e.g., rheumatoid arthritis, sarcoidosis, ulcerative colitis/inflammatory bowel disease) can have recurrent lung infections and may have concurrent bronchiectasis.^44, 45^ As shown in Table 1, the prevalence estimates for concurrent bronchiectasis in those with non-pulmonary diseases can range from 1 to 2% for inflammatory bowel disease to as high as 6% for rheumatoid arthritis.

### Referral to secondary/specialist care

Most patients with a diagnosis of bronchiectasis will be referred to secondary/specialist care for an assessment even if care is subsequently maintained by the primary care team. Specialist care may be necessary to investigate and confirm underlying causes, as specific treatment plans, including antibiotics, canvary depending on the underlying cause. Specialists will perform and/or interpret tests that may not be available in primary care, such as HRCT in some places, immunoglobulin classes (IgG, IgA, IgM, Total IgE), IgE specific to *Aspergillus fumigatu*s and *Aspergillus precipitins*.^30^ More specialised testing in certain populations, such asα_1_-antitrypsin testing,^5^ functional antibody responses to vaccination or a myeloma screening also might be performed. Testing concentrations of nasal nitric oxide^20^ to exclude ciliary dyskinesia may be required, but is generally available only at specialist centres.^46^ Adult diagnosis of cystic fibrosis may be made in patients presenting with apparent ‘non-CF’ bronchiectasis. The diagnosis should be suspected in patients <50 years of age with *P. aeruginosa* or *S. aureus* infection, male infertility or other extrapulmonary features. Sweat test and/or genotyping for common cystic fibrosis transmembrane conductance regulator mutations should be performed in these patients.^47–50^

#### Determining severity of disease

Bronchiectasis has a highly variable impact on patients. Patients with mild disease, as defined by a lower number and intensity of symptoms, may not produce sputum except during exacerbations, and will be in otherwise good health. Sputum in patients with mild disease is often mucoid (white or clear), sputum cultures are negative, and lung function is well preserved. Patients with moderate disease often have persistent symptoms in spite of standard care and may require antibiotic therapy between exacerbations. In contrast, patients with severe disease typically will have large volumes of purulent sputum even when ‘well’, have reduced lung function and frequent exacerbations and have sputum cultures positive for a range of bacteria including *P. aeruginosa*.

Exacerbations have a severe impact on quality of life.^51^ In addition, the presence of bronchiectasis is associated with an increase in mortality compared with the general population.^13^ Care decisions should be based on identifying patients at high risk of frequent exacerbations, hospital admission and death. Hospitalisation is recommended in patients with breathlessness, circulatory failure/cyanosis, hypoxia, temperature ≥38°C (100.4°F), requirement for intravenous therapy or massive haemoptysis.^2^

The bronchiectasis severity index has recently been developed and extensively validated and may enhance the ability of physicians to predict outcomes and better manage patient care.^40^ The index uses clinical data (age, body mass index, FEV_1_%, hospitalisation for severe exacerbations, exacerbation number per year, Medical Research Council dyspnoea score, *P. aeruginosa* colonisation, colonisation with other organisms and radiological severity [≥3 lobes involved]) to derive a numerical score (see the online tool: http://www.bronchiectasisseverity.com). Bronchiectasis severity index scores can be used to predict the likelihood of one or more of the major consequences of the disease: mortality, frequency of exacerbations, hospital admissions and deterioration of health-related quality of life. The FEV1, age, colonisation, extension on CT and dyspnoea (FACED) score also has been developed specifically to predict mortality in patients with bronchiectasis and is based on five clinical variables (FEV_1_%, age, *Pseudomonas* colonisation, radiological severity [≥2 lobes involved] and Medical Research Council dyspnoea score).^52^ Both scoring systems predict long-term mortality, but FACED does not reliably reflect severity of disease in terms of exacerbations and quality of life.^53–55^ Those at highest risk require specialist care and intensive follow-up and/or aggressive therapy. Neither of these scoring systems has been developed for use in primary care, and both require steps such as evaluating lobar involvement on CT that are not commonly performed during primary care assessment. Formal scoring can be useful for clinical management, but at a minimum we would recommend that primary care physicians are aware that frequent exacerbations, hospital admissions, lower FEV_1_%, significant breathlessness and the presence of *P. aeruginosa* and other pathogens are markers of worse prognosis.

## Overall goals of treatment

**Fig. 3:**
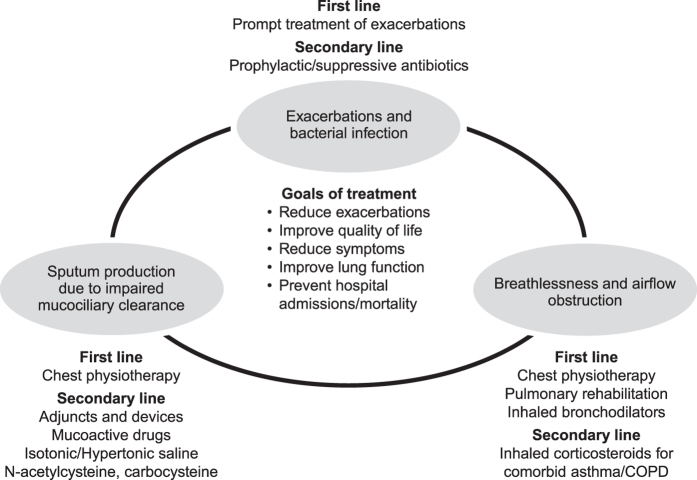
Bronchiectasis disease management. COPD, chronic obstructive pulmonary disease

The main goals of treatment are to reduce exacerbations, preserve lung function and improve the patient’s quality of life. A disease management schematic is shown in Fig. 3. Patients with bronchiectasis should be instructed in how to improve airway clearance using physiotherapy techniques at home or at a physiotherapy clinic. Treatments, both pharmacological and non-pharmacological, should focus on reducing inflammation and preventing exacerbations. Because bronchiectasis involves a permanent change in lung structure, the condition is chronic and the patient’s quality of life may be severely impacted. Care is best managed by a stepwise multidisciplinary team approach, including respiratory/chest physiotherapy.^5^

### Primary care management

Some management principles are common to all patients with bronchiectasis and include the requirement for good education about the disease, annual vaccination against influenza and vaccination against *S. pneumoniae*. Patients that are breathless will benefit from pulmonary rehabilitation as is the case with other respiratory disorders, and patients should be encouraged to exercise. Smoking cessation should be strongly advocated for the minority of patients that continue to smoke (up to 18% of patients with bronchiectasis are reported to be current smokers).^40^

Specific management strategies for bronchiectasis depend on the underlying severity and cause of disease. Patients with mild or moderate bronchiectasis are often managed and monitored in the primary care setting. Patients with mild bronchiectasis usually will not require prophylactic antibiotic therapy, but those with sputum production should perform daily physiotherapy between and during exacerbations.^5^ Patients with moderate bronchiectasis patients will typically have persistent symptoms in spite of standard care and may require prophylactic long-term antibiotic therapy as well as adjunctive treatments and strategies to promote mucus clearance (see sections below on Secondary care management and Chest physiotherapy/respiratory therapy).^5^

In the primary care setting, management should focus on monitoring the disease and implementing techniques and procedures to minimise disease progression and maximise equality of life. Regular clinic visits will allow the primary care provider to coordinate care with specialists and refer the patient to the appropriate specialist, as needed. Key considerations for the primary care provider to assess at each visit are outlined in Table 3. Regular tracking of exacerbations and symptoms allows the patient to learn more about managing his or her disease and helps to keep the care plan up-to-date. Regular monitoring of sputum^56^ (ideally twice per year) will identify the emergence of new pathogens or the development of antibiotic resistance. The previous sputum results can be used to guide future antibiotic use for exacerbations.

**Table 3:** Key questions to ask at each visit

Key points and questions for each visit
Are disease symptoms controlled?^a^
Is the patient performing pulmonary physiotherapy?
What is the frequency of exacerbations?
How are we going to treat the next exacerbation (requires recent sputum sample and knowledge of antibiotic allergies/sensitivities)?
Treatment should be based on previous sputum culture
Important to send an additional sputum sample for analysis at the start of an exacerbation
Treat for 14 days
When was last time that sputum was analysed (twice per year is recommended)?^2^
Positive culture for *Pseudomonas aeruginosa,* particularly for a new finding, should prompt review and often referral to secondary care
Are there any signs of deterioration?

^a^Key symptoms are cough, sputum production and breathlessness

Spirometry is a key pulmonary function test used to assess lung function and should be performed at least annually, and preferably at each clinic visit in patients with severe bronchiectasis.^2, 5, 10, 25, 31^ Significant worsening over time in pulmonary function indicates worsening disease and should drive an intensification in treatment.

The discussion between the primary care provider and patient during these routine visits provides an opportunity for ongoing patient education, a key component of disease management. Educating the patient regarding the disease will ensure that he or she understands the clinical approach and management plan, including the importance of sputum analysis.^2^ It is crucial that patients are able to recognise an exacerbation and how best to access the medical care team when necessary. Knowledge of airway physiotherapy/airway clearance techniques will help reduce the impact of exacerbations and improve quality of life. In a study to determine compliance with a bronchiectasis treatment program in 75 patients, only 53% were found to be compliant with medical treatment and only 41% were compliant with airway clearance techniques.^57^ Improving compliance with all aspects of recommended therapy is, therefore, a key goal of enhanced patient education. Indeed, the benefits of patient education have been demonstrated by expert patient self-management programs that promote action planning, role modelling, problem solving, reinterpretation of symptoms and decision making.^58^

#### Exacerbation monitoring/management

An exacerbation can be defined as a significant worsening of symptoms over several days, which may include an increase in the frequency of cough, shortness of breath, increase of sputum volume, viscosity and/or purulence.^2^ In the outpatient setting, assessments of exacerbations should include the history, a clinical examination, a sputum sample for culture prior to beginning antibiotic treatment and a review of previous sputum microbial analyses.^2^

Medications should be chosen based on current and previous bacteriological results.^2, 5, 31^ For exacerbations that are not severe, oral antibiotics are appropriate. Standardised courses of antibiotics(14 days) are recommended for all patients with exacerbations owing to the higher airway bacterial loads observed in bronchiectasis. Antibiotic prescribing is variable in the United States and among European countries.^59–61^ Recent guidelines suggest that coordinated efforts to develop antibiotic stewardship programs can help to minimise the development of antibiotic resistance.^59, 60^

Patients requiring intravenous antibiotics include those with severe infections requiring hospital admission, patients with organisms resistant to oral antibiotic agents (most frequently *P. aeruginosa* resistant to ciprofloxacin or other multi-drug resistant Gram-negative organisms) or patients who have failed to improve with 14 days of targeted oral antibiotics. Patients with respiratory failure, confusion, haemodynamic instability or large volume haemoptysis will require admission to hospital. Small volume haemoptysis is relatively common in bronchiectasis and may simply require antibiotic therapy. Patients experiencing haemoptysis for the first time should be evaluated by a specialist and patients with large volume haemoptysis (e.g.,>100 ml), or haemoptysis with hypoxaemia or haemodynamic instability should be referred to hospital.

Although most often administered to hospitalised patients, intravenous antibiotics have been shown to be effective and safe when administered at home, after proper instruction.^62^

#### *P. aeruginosa* eradication

*P. aeruginosa* is a special case because of its strong impact on prognosis. When *P. aeruginosa* is isolated for the first time in patients with bronchiectasis most international guidelines recommend attempting to eradicate the organism when isolated for the first time in sputum. Our recommendation for primary care is to send sputum samples for stable patients at least once per year, and preferably more often. In the event of a first positive sample for *P. aeruginosa,* patients should send a further sample for culture, and treat with oral ciprofloxacin 750 mg twice daily for 14 days. A repeat sputum sample should be sent after antibiotics to determine if the treatment has been successful and the patient should be referred to a specialist who will determine whether to add intravenous or inhaled antibiotics to the regime.^2^

#### Inhaled bronchodilators and corticosteroids

Although there is limited evidence, it is reasonable to treat patients with significant breathlessness with inhaled bronchodilators, such as combined long-acting β-agonists and anti-muscarinics. This is particularly the case when airflow obstruction is present.

Recent recommendations suggest no role for inhaled corticosteroids in bronchiectasis unless the patient has coexisting asthma or COPD.^2, 5, 31^ In a recent reviews, these agents have not been shown to have significant beneficial effects on lung function or exacerbation frequency in bronchiectasis patients without asthma or COPD.^5, 51^

### Secondary care management

Once a patient is diagnosed, he or she should be referred to secondary care for assessment and to help determine the underlying cause of the disease as described in the Introduction. Secondary care physicians in most countries will provide the initial disease education and provide access to chest physiotherapy.

Patients with immune deficiency can often be treated by immunoglobulin replacement therapy under the care of an immunologist.^2^ Specialists should also be involved in the treatment of patients with ABPA, who will nearly always have asthma, elevated total and *Aspergillus-*specific IgE and IgG-mediated immunological responsiveness. Treatment of ABPA involves prolonged treatment with oral corticosteroids. Antifungal agents may also be used as steroid-sparing agents.^2, 5, 63^ Secondary care teams are necessary when managing patients with severe bronchiectasis requiring long-term oxygen therapy and/or persistent symptoms requiring oral and/or inhaled antibiotics.^5, 30^ Specialist care is also required if chronic *P. aeruginosa*, opportunist mycobacteria (NTM), methicillin-resistant *S. aureus* colonisation or ABPA occur,^2, 5, 30^ and for patients with deteriorating lung function, as treatment for these conditions will often require specialised combinations of antibiotics/antifungals and/or robust monitoring.^2, 63^ Some aetiologies, such as rheumatoid arthritis and PCD, are associated with a more severe course and these patients will usually be managed in secondary care.

Recent controlled trials have provided evidence that macrolide antibiotics (azithromycin, erythromycin) can reduce exacerbation frequency and improve quality of life.^5, 51, 63, 64^ It is important to note, however, that long-term treatment can lead to the development of antibiotic resistance.^64^ As use of long-term macrolides is increasingly common, it is important for primary care physicians to be aware of the potential complications and consequences of macrolide treatment. Up to 20% of patients will develop gastrointestinal side effects with macrolides,^65, 66^ and this is more common if higher doses are used. If this becomes troublesome, a dose reduction or change to an alternative oral antibiotic may be needed. Macrolides can cause hearing loss^67^ and this may initially present with tinnitus. This is usually reversible but macrolides should be discontinued immediately if tinnitus is reported. Macrolides can prolong the QT interval^68^ and so should not be co-prescribed with other drugs that prolong the QT interval.

Inhaled formulations of antibiotics deliver higher concentrations of a drug to sites of infection within the airway than with delivery by oral or intravenous routes. A meta-analysis of nine trials indicated that inhaled formulations reduced sputum bacterial load, increased the eradication of *P. aeruginosa*, reduced exacerbations and decreased health-care utilisation.^69^ It must be emphasised, however, that as yet, no inhaled antibiotics have been approved by regulatory agencies for treatment of bronchiectasis. Several inhaled antibiotics are licensed for cystic fibrosis bronchiectasis, but use of data from antibiotic trials in patients with cystic fibrosis are not always directly translatable to non-cystic fibrosis bronchiectasis. The most common adverse effect of inhaled antibiotics is bronchospasm. As a result, it is recommended that inhaled antibiotics are always initiated in secondary care, and a test dose is administered in a controlled environment (e.g., hospital ward or outpatient clinic setting) to ensure the patient does not experience an adverse reaction.

#### Chest physiotherapy/respiratory therapy

Physiotherapy techniques are recommended as non-pharmacological methods for mucus clearance, and should be tailored to the individual patient through input from a specialist in physiotherapy where possible. The most frequently used technique in Europe is the active cycle of breathing technique (ACBT).^70^ In a systematic review and meta-analysis, ACBT was shown to be more effective in reducing sputum volume than other methods.^71^ ACBT includes breathing control (tidal breathing at a normal rate), thoracic expansion exercises (deep breathing exercises), forced expiration of 1 or 2 huffs, followed by more breathing control. The huffs assist in clearing secretions in the larger upper airways. These methods can be viewed online by searching www.youtube.com for ‘ACBT breathing technique.’ Several excellent physiotherapist-narrated videos are available demonstrating this technique.

Several oscillatory positive expiratory pressure devices are also available to assist with airway clearance, including the Flutter, Shaker and Acapella, which are handheld devices that use exhaled breath to create oscillating positive expiratory pressure to help clear mucus.^72, 73^ The Lung Flute is a small self-powered handheld audio device that produces a low frequency acoustic wave with moderately vigorous exhalation, rather than oscillatory back pressure, to increase mucus clearance.^74^ The majority of patients can manage their chest clearance without requiring devices, but these may be helpful as adjuncts under the supervision of specialist chest physiotherapists. It is recommended that patients with severe symptoms, frequent exacerbations or those experiencing difficulty with expectoration are reviewed regularly by a specialist physiotherapist.

#### Surgical options

Removal of portions of damaged lung may be considered in patients with severe bronchiectasis who have failed medical therapy.^2^ In patients with localised disease, recent reviews suggest that the removal of the permanently damaged areas of the lung can result in significant symptom resolution and an improved quality of life.^75, 76^ In a meta-analysis of 38 studies covering 5541 patients who had surgical resection for management of bronchiectasis, operative morbidity and mortality rates were 16.7% and 1.5%, respectively.^76^

### Treatment algorithm for the deteriorating patient

**Fig. 4:**
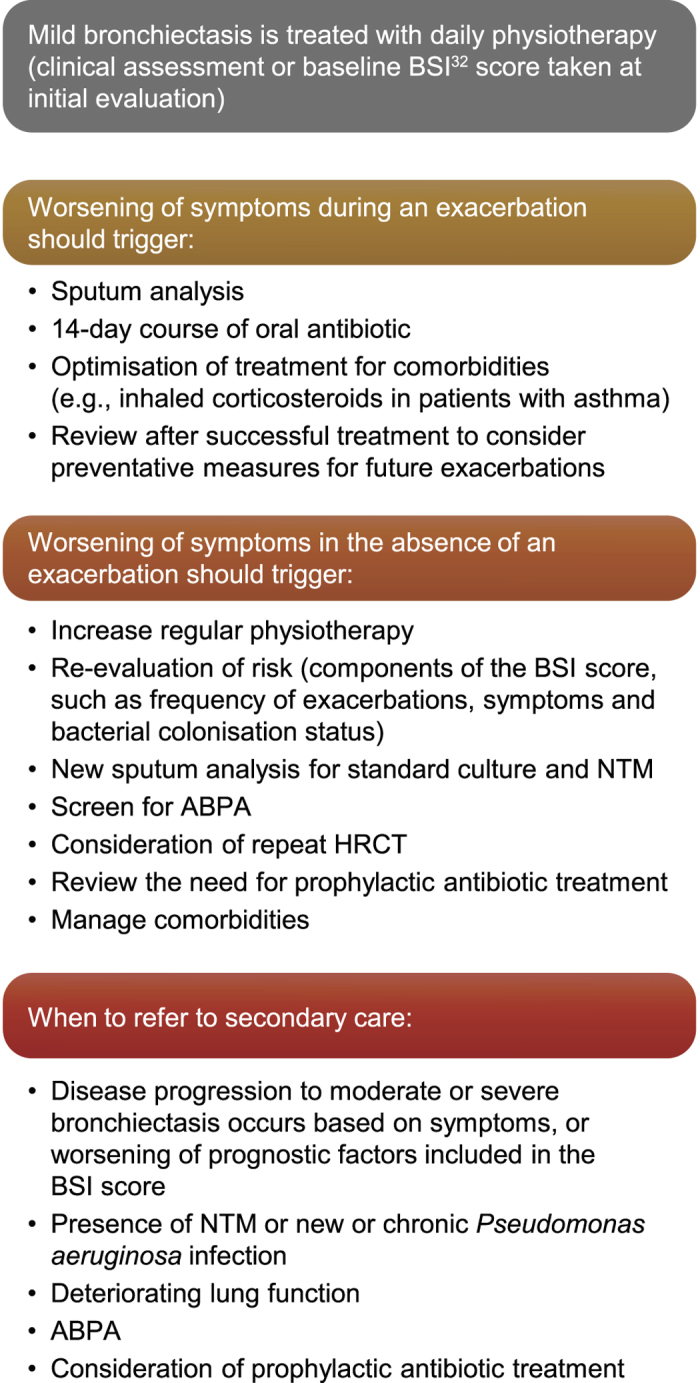
Considerations for the management of bronchiectasis in a patient with worsening symptoms in primary care. ABPA, allergic bronchopulmonary aspergillosis; BSI, bronchiectasis severity index; HRCT, high-resolution chest computed tomography; NTM, non-tuberculous Mycobacteria

A major challenge in the care of patients with bronchiectasis in the primary care setting is managing those whose condition is deteriorating. This situation may require input from an expanded team of specialists to intensify and refine the treatment plan. Figure 4 illustrates an algorithm for advancing the treatment plan for typical patient scenarios of deterioration in their condition, which reflects the authors’ clinical experiences. Deterioration is defined as an increase in the number of exacerbations (>2 per year), hospital admissions, rapidly declining FEV_1_ and new isolation of *P. aeruginosa* in sputum associated with worsening of symptoms. It is essential for primary care physicians to be confident in identifying patients such as those that require referral or re-referral to secondary care and intensified therapy.

## Conclusions

Bronchiectasis is a complex chronic disease, often resulting in disability and impaired quality of life. Patient education and compliance with care providers’ recommendations are key to successful disease management. Progress in determining best practices and treatments will be aided by patient recruitment into recently developed patient registries in the United States and Europe.^5, 51^ These registries will encompass the experiences of many more and varied patients than can be included in individual clinical trials. They encourage international collaborations and can help drive research with the overall goal of improving clinical care.^51^

Although mild and moderate bronchiectasis usually can be managed in the primary care setting, collaboration with specialists to develop an individualised patient management plan would provide advanced planning should the patient’s condition worsen. Those with more advanced disease who require long-term antibiotic therapy should be referred to secondary care.
